# Hot and cold flavors of southern California’s Santa Ana winds: their causes, trends, and links with wildfire

**DOI:** 10.1007/s00382-021-05802-z

**Published:** 2021-05-31

**Authors:** Alexander Gershunov, Janin Guzman Morales, Benjamin Hatchett, Kristen Guirguis, Rosana Aguilera, Tamara Shulgina, John T. Abatzoglou, Daniel Cayan, David Pierce, Park Williams, Ivory Small, Rachel Clemesha, Lara Schwarz, Tarik Benmarhnia, Alex Tardy

**Affiliations:** 1 Scripps Institution of Oceanography, University of California San Diego, La Jolla CA, USA; 2 Desert Research Institute, Reno NV, USA; 3 School of Engineering, University of California Merced, Merced CA, USA; 4 Lamont Doherty Earth Observatory, Columbia University, Palisades NY, USA; 5 U.S. National Weather Service, San Diego CA, USA; 6 School of Public Health, University of California San Diego, La Jolla CA, USA

## Abstract

Santa Ana winds (SAWs) are associated with anomalous temperatures in coastal Southern California (SoCal). As dry air flows over SoCal’s coastal ranges on its way from the elevated Great Basin down to sea level, all SAWs warm adiabatically. Many but not all SAWs produce coastal heat events. The strongest regionally averaged SAWs tend to be cold. In fact, some of the hottest and coldest observed temperatures in coastal SoCal are linked to SAWs. We show that hot and cold SAWs are produced by distinct synoptic dynamics. High-amplitude anticyclonic flow around a blocking high pressure aloft anchored at the California coast produces hot SAWs. Cold SAWs result from anticyclonic Rossby wave breaking over the northwestern U.S. Hot SAWs are preceded by warming in the Great Basin and dry conditions across the Southwestern U.S. Precipitation over the Southwest, including SoCal, and snow accumulation in the Great Basin usually precede cold SAWs. Both SAW flavors, but especially the hot SAWs, yield low relative humidity at the coast. Although cold SAWs tend to be associated with the strongest winds, hot SAWs tend to last longer and preferentially favor wildfire growth. Historically, out of large (> 100 acres) SAW-spread wildfires, 90% were associated with hot SAWs, accounting for 95% of burned area. As health impacts of SAW-driven coastal fall, winter and spring heat waves and impacts of smoke from wildfires have been recently identified, our results have implications for designing early warning systems. The long-term warming trend in coastal temperatures associated with SAWs is focused on January–March, when hot and cold SAW frequency and temperature intensity have been increasing and decreasing, respectively, over our 71-year record.

**Supplementary Information:**

The online version contains supplementary material available at 10.1007/s00382-021-05802-z.

## Introduction

**Fig. 1:**
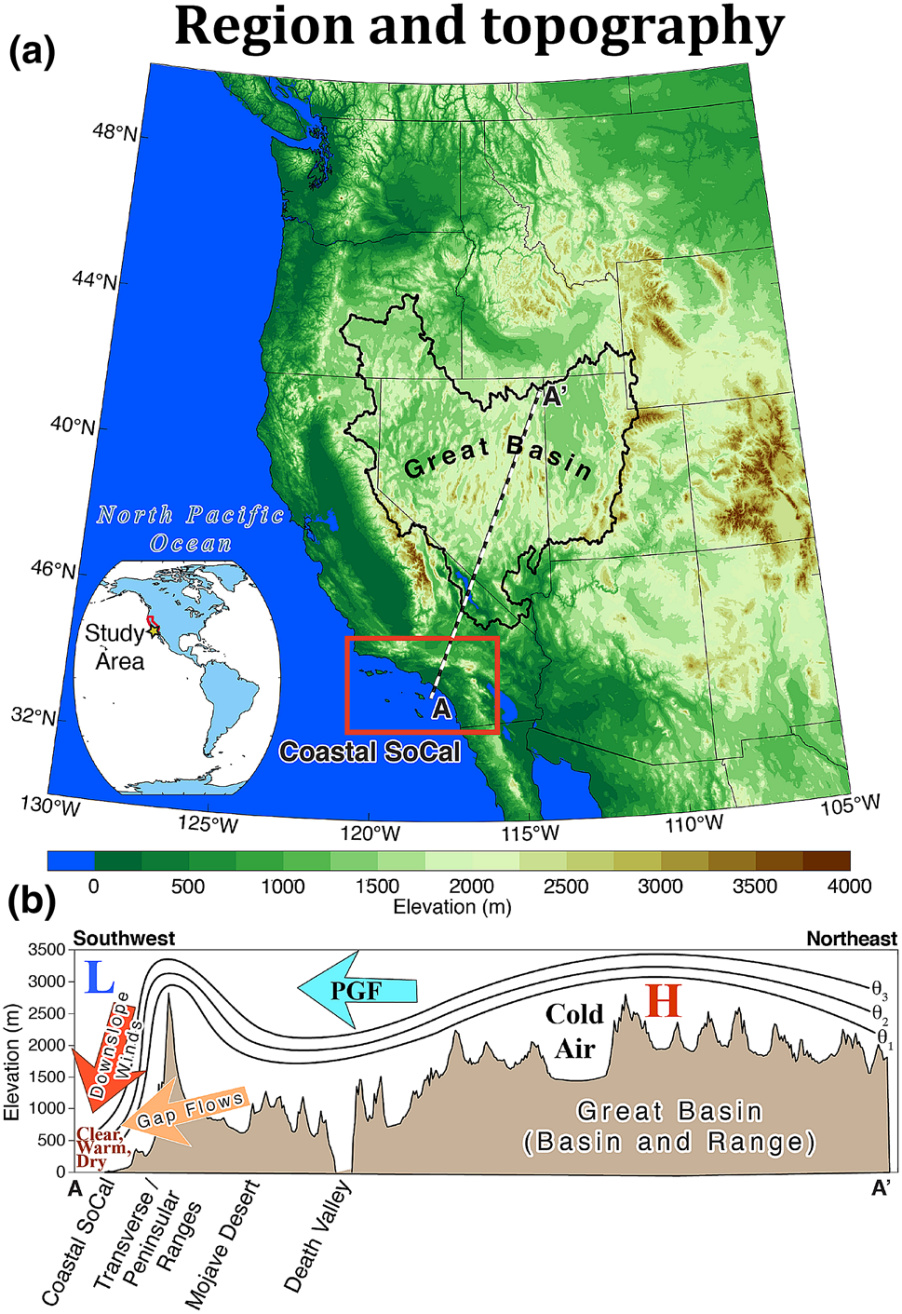
Geographic setting and topography of the western United States (**a**) along with cross sectional topography (**b**) along the transect indicated in a dashed line on the map with point A marking the coastal outflow region of SAWs and point A’ marking the northernmost extent of the Great Basin—the region of origin for air masses feeding the SAWs

The Santa Ana winds (SAWs) of Southern California (SoCal) are notorious for spreading catastrophic wildfires (Moritz et al. 2010) and influencing air quality (Aguilera et al. 2020). However, SAWs are also known to produce extreme heat narrowly focused along the densely populated coastal zone (Gershunov and Guirguis 2012; Clemesha et al. 2017). The Great Basin—a high inland desert at an elevation of > 1200 m (Fig. 1)—is the source region for air masses implicated in SAW, that are driven by a regional pressure gradient force (PGF) between the Great Basin and offshore of California (Hughes and Hall 2010; Abatzoglou et al. 2013). Often associated with amplified anticyclonic flow aloft (Hatchett et al. 2018), the lower tropospheric PGF drives northeasterly winds that warm via adiabatic compression as air flows from the elevated terrain of the Great Basin over the 3000 m Transverse and lower Peninsular ranges to reach maximum temperatures at sea level (Fig. 1). The cooler and denser Great Basin air relative to the maritime airmass over California promotes acceleration of the wind over the lee-slopes of coastal topography. Local and regional variation in SAW results from the range of downslope windstorm mechanisms involved, including strong cross-mountain flow and varying atmospheric stability structures (Durran 1990; Hughes and Hall 2010; Cao and Fovell 2016; Abatzoglou et al. 2021) in addition to katabatic (Hughes et al. 2011; Kolden and Abatzoglou 2018) or gap flow (Huang et al. 2009) components of terrain-forced downslope winds.

While the typical lower tropospheric regional PGF (Fig. 1) is most frequently established during winter and the coldest Great Basin airmasses occur when nights are longest in December and January, these two main SAW ingredients can co-occur any time from early fall to late spring. This determines the SAW season (Guzman-Morales et al. 2016). In early fall (September–October) and occasionally in late spring (April–May), when Great Basin temperatures are only moderately cool and synoptic pressure gradients develop, SAWs can cause record-breaking coastal heat waves as air descends at the dry adiabatic rate (9.8 °C per 1000 m). Many of the all-time heat records at coastal SoCal locations were registered during fall with the first SAWs of the season. For example, on September 25, 1978, SAW drove temperatures to 40.5 °C in downtown Los Angeles.^1^ Even in winter, SAWs can result in anomalous coastal heat that can catch vulnerable communities off-guard. On February 27, 2020, for example, Los Angeles International Airport recorded 29.4 °C with 30.6 °C recorded at Camarillo Airport,^2^ while 17.9 °C is the normal maximum temperature for SoCal’s coastal zone for this day. As we shall see below, over the last 71 years of record, two of the ten all-time high maximum temperatures in SoCal’s coastal zone were associated with SAWs.

Surface maximum temperatures are further increased by intense insolation under clear skies and the presence of a dry airmass. Additionally, synoptic wave breaking and isentropic drawdown induced from terrain-influenced circulation further warm the surface (Kaplan et al. 2017). Such conditions of warm, dry winds and strong solar heating are emblematic of SAWs. Offshore winds and solar heating are especially important for promoting anomalous heat in spring, when coastal sea surface temperatures are seasonably cool (~ 13 °C) and persistent coastal low-level cloudiness otherwise cools SoCal’s coastal zone (Clemesha et al. 2016; Iacobellis and Cayan 2013). SAWs also have important societal impacts including on public health. Off-season excessive heat has been linked to premature mortality in coastal SoCal (Kalkstein et al. 2018). Schwarz et al. (2020) tied heat-health hospitalizations directly to SAW events; these impacts may be worsened by smoke when SAWs fan wildfires (Aguilera et al. 2020). Many coastal communities are composed of vulnerable populations with reduced adaptive capacity, e.g. no air conditioning (Guirguis et al. 2018), further exacerbating heat-related SAW impacts.

In addition to impactful heat, SAWs can produce extremely cold conditions along the coast. Cold SAWs have been documented in the popular press.^3^ As we show, many of the absolute coldest days on record occurred during or directly preceding SAWs. To our knowledge, hot and cold “flavors of SAWs” have not been documented in the academic literature. We therefore investigate both flavors of SAWs to see what, if any, dynamical differences exist between cold and hot SAWs. We initially hypothesized that GB snow cover promotes cooler airmasses that favor cold SAWs. Yet we find that snow is only part of an intricate reality, which includes fundamentally different synoptic setups resulting in hot and cold flavors of SAWs. Improved understanding of SAW flavors will result in more accurate determination of wildfire risk, more skillful predictions at longer lead times, better warnings for impact-based decision support (Uccellini and Ten Hoeve 2019), more useful climate change projections, and improving resilience to the greatest impacts of SAWs, including public health and safety via thermal extremes and wildfire.

Multi-decade SAW climatologies have been constructed and analyzed for climate-scale behavior (Abatzoglou et al. 2013; Guzman Morales et al. 2016—hereafter GM’16, Rolinski et al. 2019). Focusing mainly on wind, these studies identified patterns of climate variability in SAW activity, highlighting regional climate forcings including El Niño–Southern Oscillation (ENSO) and the Pacific Decadal Oscillation (PDO). Climate change is expected to diminish SAW activity (Hughes et al. 2011) by eroding SAW frequency in the early and late season (Guzman Morales and Gershunov 2019, hereafter GMG’19) with trends projected to emerge early in the twenty-first century. SAW-driven coastal temperature anomalies have not been studied and their climatology has not been assembled. As the Great Basin is projected to warm more rapidly than the coastal zone (Cayan et al. 2013), we expect SAW-driven coastal temperature extremes may warm at a greater rate than the background climate. It is thus timely to understand the past, current, and future behavior of Santa Ana winds as well as the compound impacts they generate via wildfires (Small 1995; Westerling et al. 2004; Moritz et al. 2010; Rolinski et al. 2016; Kolden and Abatzoglou 2018), air quality (Delfino et al. 2009; Leibel et al. 2019; Aguilera et al. 2020a, b), and temperature extremes (Schwarz et al. 2020) on coastal SoCal—a marine-influenced, densely populated region where public health is acutely impacted by heat (Guirguis et al. 2014, 2018) and wildfire smoke (Aguilera 2021a; b). Our goal here is to understand and describe hot and cold flavors of SAWs, their historical climate-scale behavior, their drivers, connection to wildfire, and observed trends over the past seven decades.

## Data and methods

An hourly record, spanning 1948–2012, of dynamically downscaled SAW activity on a 10 × 10 km grid was constructed, validated against the available observations, analyzed, and presented by GM’16. The SAW Regional Index (SAWRI) was also constructed for SoCal and later updated and converted to daily values using a hybrid dynamical-statistical downscaling of the NCEP/NCAR Reanalysis 1 (Kalnay et al. 1996) (R1D-SAWRI) by GMG’19. The version of R1D-SAWRI used here constrained the number of SAW days by spatial extent filtering. Only days with local (grid-wise) conditions in at least ~ 60% of the SAW domain were considered SAW days. R1D-SAWRI spans January 1948 to December 2018 (71 years).

**Fig. 2:**
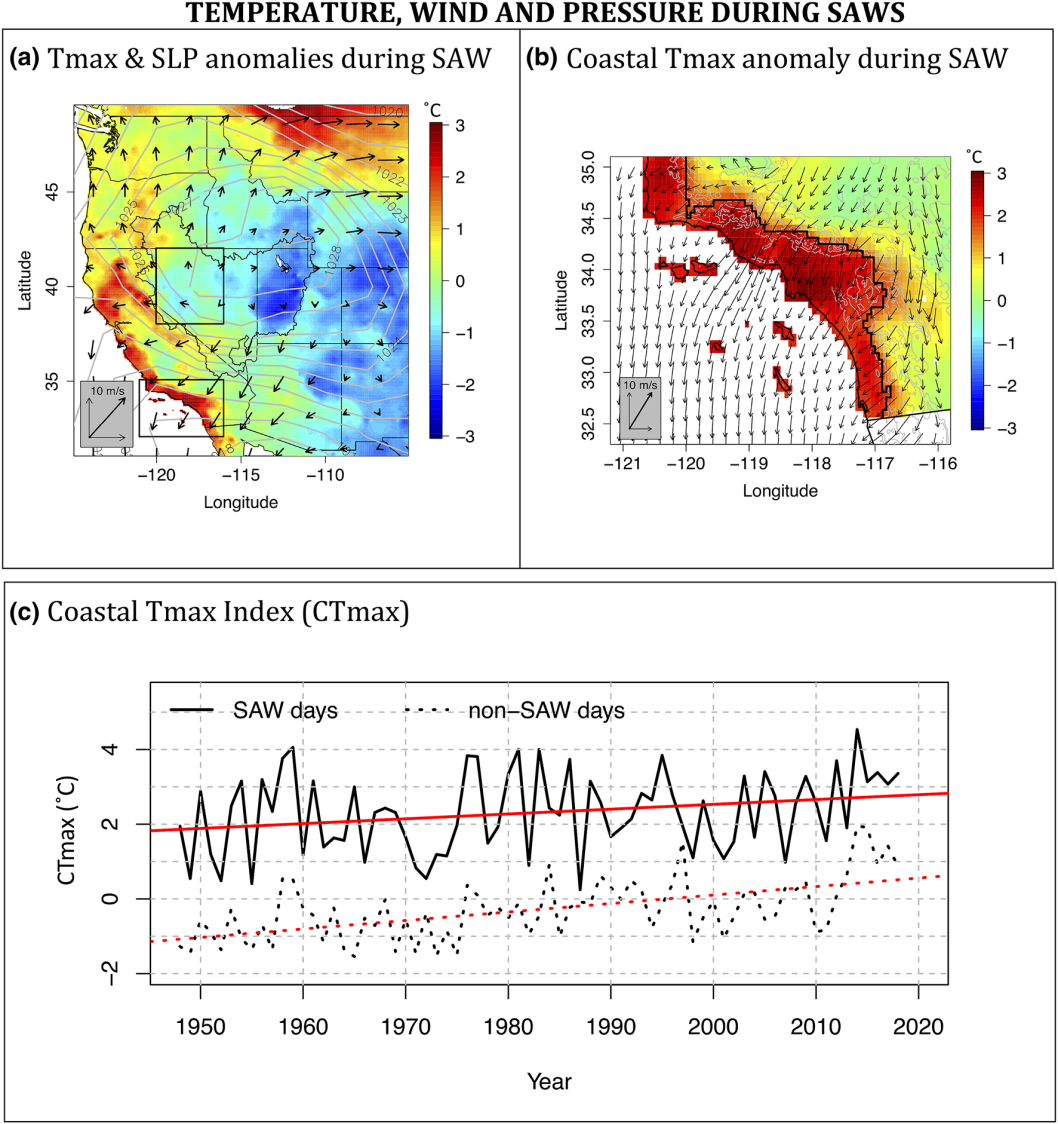
Tmax anomalies during SAW days from 1948 to 2018. **a** Shows composites of Tmax anomaly (color shades), SLP (contours) and 10 m R1 wind field (black arrows) over the larger Western US region. The SoCal and western Great Basin domains, considered in this study, are shown in black boxes. The full extension of the Great Basin, as shown on **a**, is added for reference. **b** Enlarges the SoCal region, inset on **a**, and shows composites of Tmax (color shades) and downscaled wind fields (black arrow). The region delineated with thick black lines corresponds to the warmest 20% (Tmax > 1.8 °C) of local temperature anomalies due to SAW conditions (coastal SoCal). **c** Shows the annually- and spatially-averaged coastal Tmax index (CTmax) for SAW (solid) and non-SAW (dotted) days with fitted linear trends, which are significant with 95% confidence at 0.13 and 0.23 °C/decade, respectively for SAW and non-SAW days. Correlation between SAW days and non-SAW days CTmax time series is 0.46

We use observed daily maximum and minimum temperature (Tmax and Tmin, respectively) data from First Order and Cooperative Observer meteorological observations interpolated onto a 6 × 6 km grid using inverse distance weighting of the four nearest stations to each grid cell, down-weighting stations close to other stations, and applying a fixed lapse rate for interpolating in complex topography (Livneh et al. 2013, 2015). Tmax and Tmin anomalies were computed relative to their seasonal cycle at each 6 × 6 km grid cell. The seasonal cycle was modeled, separately for Tmax and Tmin, via double (annual and semi-annual) harmonics fitted to daily temperatures and regressed out of the daily temperature data (Gershunov and Roca 2004). Following GM’16 and GMG’19, we used SAWRI to identify and quantify the pattern of coastal warming due to SAWs (Fig. 2a, b). A pattern of anomalous warming associated with all SAW events is readily apparent along the low-elevation coastal zone. The region corresponding to the warmest 20% (Tmax > 1.8 °C) of local temperature anomalies due to SAW conditions is the region we refer to as coastal SoCal (delineated by black lines in Fig. 2b). We isolate and delineate this spatial pattern via the + 1.8 °C anomaly isotherm that corresponds to the 80th percentile of anomalous Tmax average during SAW days over the coastal SoCal domain.

The coastal de-seasonalized temperature indices (CTmax and CTmin) for SAW and non-SAW days were constructed daily by spatial averaging of Tmax and Tmin anomalies over the coastal zone delineated in Fig. 2b and over all SAW and non-SAW days of the SAW season (September–May). Note that SAWs themselves impact the CTmax and CTmin seasonal cycles, with a mean of 4 and 12 SAW days in October and December, respectively. Figure 2c presents the resulting annual time series along with fitted trend lines. Similarly, we compute analogous daily averages of temperature anomalies for the western Great Basin (GBTmax and GBTmin) defined as the square region over the Great Basin delineated on Fig. 2a—this is the primary region where the SAWs are rooted.

**Fig. 3:**
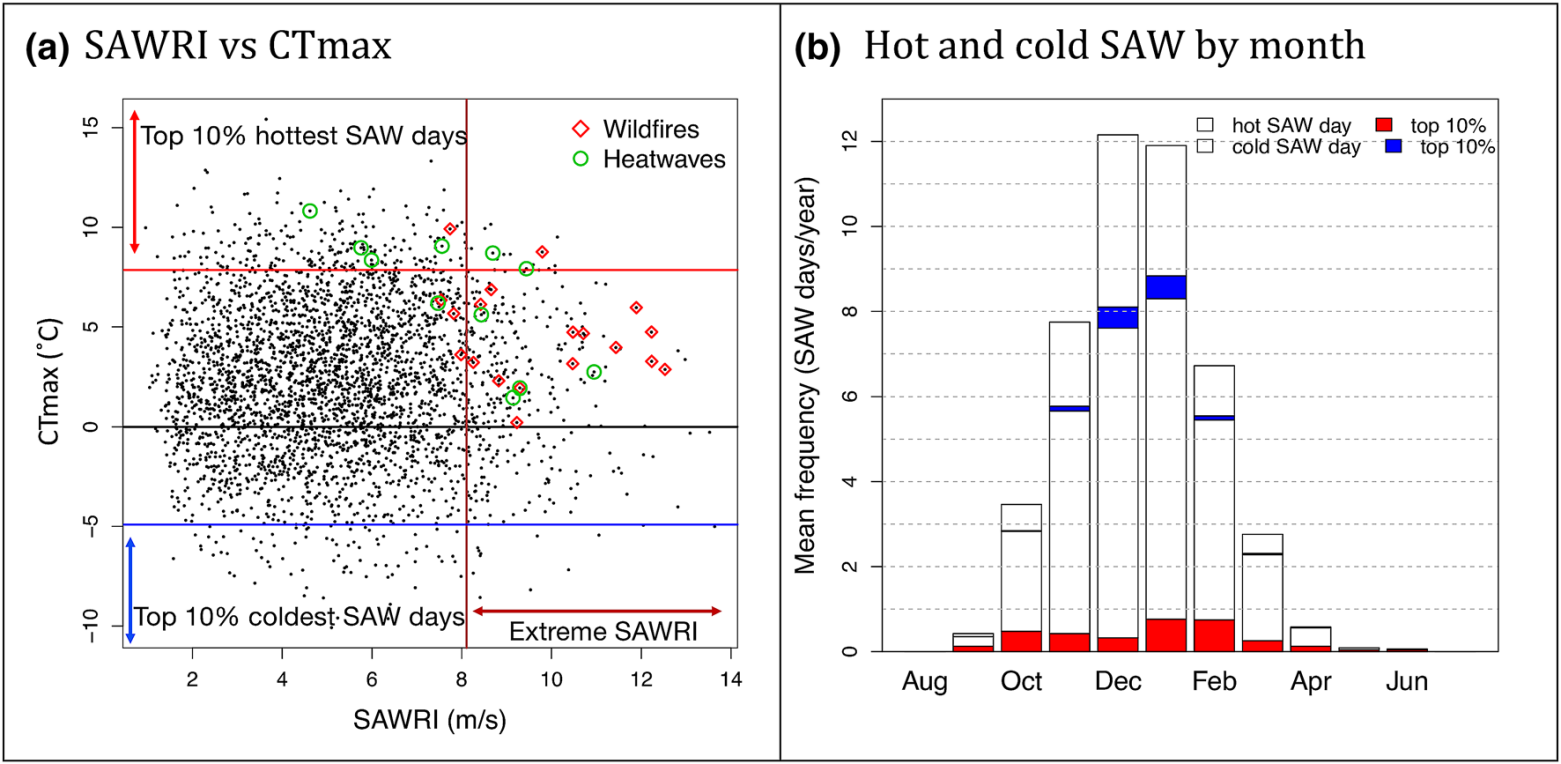
**a** Daily CTmax versus SAWRI, extreme SAWRI is depicted with a dark red line on the x-axis, and the CTmax thresholds for the top 10% hottest and coldest SAW days are marked with red and blue lines, respectively, on the y-axis. Heat waves and wildfires associated with SAW events from NWS report “A History of significant weather events in Southern California” are marked in green circles and red diamonds, respectively. **b** Mean frequency of hot and cold SAW days by month. Extreme top 10% hottest and coldest days are depicted in bright red and blue sections respectively

We also categorize SAW days into hot and cold SAW varieties based on their positive and negative CTmax values as well as the extreme 10% hottest and 10% coldest SAW days that fall above the 90th and below the 10th percentiles of CTmax, respectively (Fig. 3a). Thus all SAW days are classified as hot or cold SAWs according to whether the maximum coastal temperature anomaly is positive or negative, while the extreme hot and cold SAWs are highlighted. When analysis is performed over SAW *events* (only for duration and minimum relative humidity assessments, Figure S1), we classify hot (cold) SAW events based on their positive (negative) mean CTmax. Events with mean absolute CTmax below the 5th percentile of all event means (|CTmax|< 0.27 °C, which amounted to 5% of all SAW events) were considered undefined and were discarded from analyses focusing on SAW *events*.

Daily, gridded 4 km resolution snow water equivalent (SWE) reanalysis spanning October 1981–September 2018 (Zeng et al. 2018) was used to compute daily snow coverage for hot and cold SAW days over the western Great Basin (Fig. 2a). For each grid cell, we assign 1 and 0 snow coverage values for SWE > 0 and SWE = 0, respectively.

Additional data sets include daily precipitation data evaluated on the same 6 × 6 km grid as temperature (Livneh et al. 2013, 2015) and wildfire observations including start dates, acres burned, and fire perimeters from the California Department of Forestry and Fire Protection's Fire and Resource Assessment Program (FRAP: https://frap.fire.ca.gov/frap-projects/fire-perimeters/). We also use hybrid statistical–dynamical downscaled relative humidity data (RH) produced by a statistical downscaling model (Localized Constructed Analogs—LOCA; Pierce et al. 2014, 2015; Pierce and Cayan 2015) forced with hourly ERA5 reanalysis humidity fields. Since humidity data at the desired 3 km resolution is not readily available from observations, we trained LOCA with the relative humidity field simulated by the WRF model (Skamarock et al. 2008), resulting in a hybrid statistical–dynamical downscaling scheme covering the state of California. WRF in turn was forced with 6-h data from the National Centers for Environmental Prediction (NCEP) FNL (Final) Operational Global Analysis (NCEP 2000). The purpose of using this hybrid scheme (downscaling ERA5^4^ using LOCA trained on WRF output) rather than using the WRF output directly was to extend the length of the WRF output, which was only available over the period 2003–2018 (16 years), to the full period ERA5 is available, 1979–2019 (41 years). Our previous analyses of LOCA downscaled humidity indicates that errors in the downscaled field with respect to the training data are about 0.5% in the mean with a RMSE of about 2% (Pierce and Cayan 2015).

The National Weather Service (NWS) identified heat waves and wildfires associated with SAWs in “A History of Significant Weather Events in Southern California”. This report documents remarkable (not comprehensive) cases from 1859 to 2017, and is used here for reference purposes of our R1D-SAWRI record. We note that every Santa Ana wind event associated with wildfires or heatwaves, with the exception of a SAW-driven heatwave on 09/26/1963 that has been documented in the NWS report, is also detected by R1D-SAWRI.

## Results

### Hot and cold SAW flavors

The highest absolute average Tmax along coastal SoCal (absolute values of CTmax) typically occurs in September (Table S1a). Seven out of the ten absolute hottest CTmax days occurred in September, including the overall record heat (39.5 °C averaged over SoCal’s coastal zone on September 27, 2010), which was associated with Rossby wave breaking and terrain-induced circulations (Kaplan et al. 2017), but not with a SAW event. Two of the ten absolute warmest CTmax days on our record were associated with SAWs (Table S1a). The hottest SAW-driven CTmax occurred on September 24, 1978, trailing the overall record by 1.5 °C. Five of the top ten SAW-associated absolute hottest days occurred in September, three in October, one in April and one (the second hottest) in June of 1981—a highly unusual, extremely late-season SAW event.

The hottest SAWs, relative to seasonal normal conditions, however, tend to occur in April–May (Table S2), when they create a distinct contrast from otherwise seasonably cool temperatures associated with cool coastal sea surface temperature and the onset of coastal low-level cloud season (Clemesha et al. 2016; Iacobellis and Cayan 2013). These conditions spell a cool start to the Mediterranean dry and warm season in coastal SoCal. This seasonal coastal coolness amplifies the relative coastal warm anomalies associated with hot SAWs. Eight of the ten hottest relative CTmax days occurred in April and May; four of them were SAWs.

On the cold side of the SAW spectrum, three of the absolute coldest 10 days on record were SAW days. The latest such event occurred in December 1990 and was SAW-related, while the other nine days occurred prior to 1973. Five of the 10 coldest SAW days occurred in 1949 from three separate SAW events in early January, late January and mid-February. The coldest SAWs demonstrate a greater range of maximum wind speeds than the hottest SAWs, which tend to be on the weaker side (Fig. 3a). For both hot and cold days, whether SAWs or not, the greatest anomalies are found in CTmax rather than CTmin. We therefore focus on CTmax going forward.

A sizable minority (28%) of SAW days are anomalously cold (Fig. 3a, b). Cold SAW frequencies peak during peak SAW season in December and January when ~ 38% of all SAWs were historically cold, with a majority of extreme cold SAW days in these months (Fig. 3b). Extreme (top 10%) hot SAW days occur most frequently in January and February (~ 0.75 year^−1^) but early fall and late spring months have the highest relative proportion of extreme hot SAW days. Hot Santa Anas tend to be longer lasting (~ 4 days on average compared to ~ 3 days for the cold SAWs—Figure S1a), with slightly longer duration for extremes of both flavors. Hot SAWs also tend to be drier in terms of RH than their cold counterparts (Figure S1b).

### Synoptic determinants of hot and cold SAWs

**Fig. 4:**
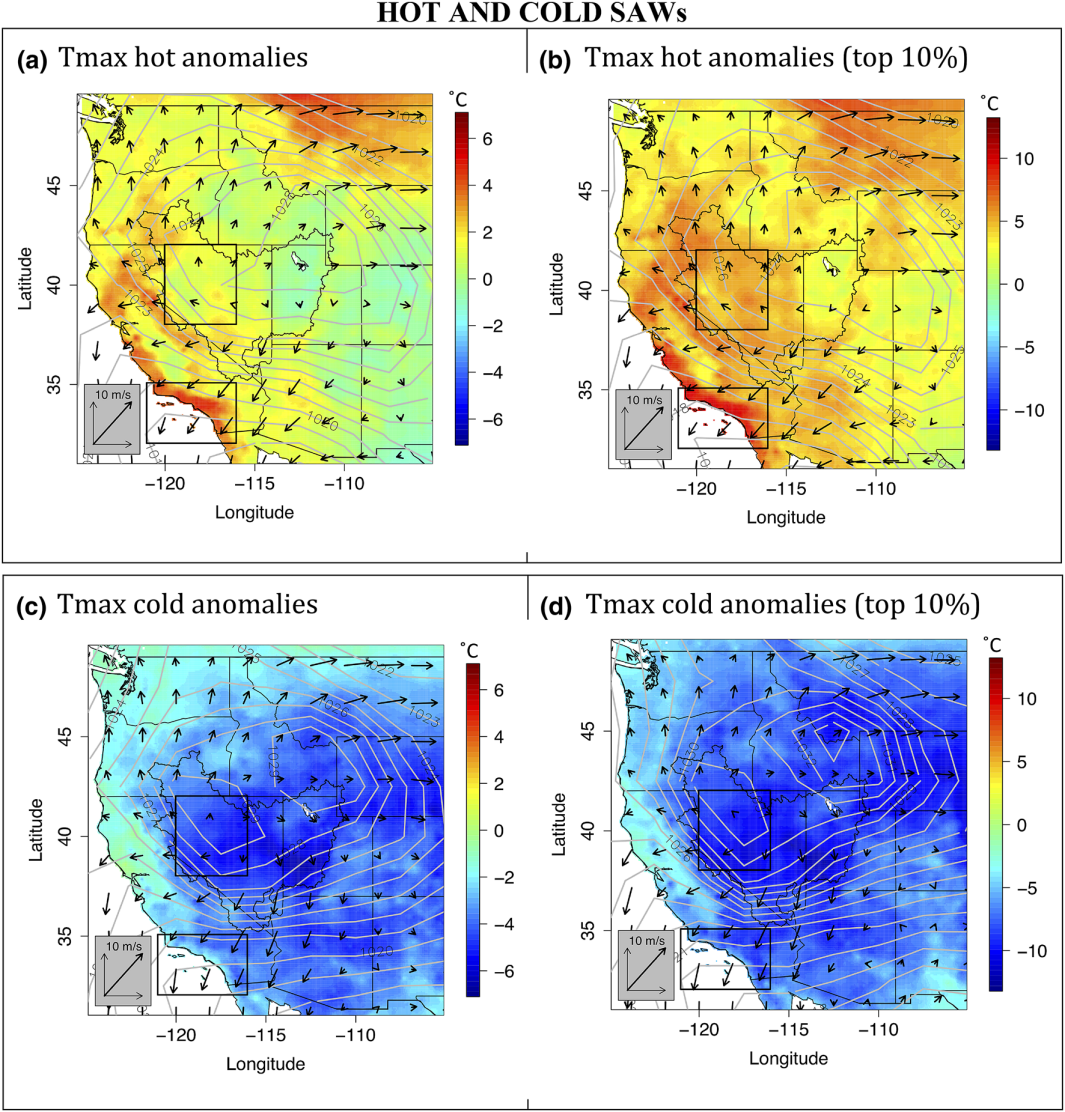
Tmax anomalies, SLP and wind field composites over the Western U.S. as in Fig. 1, but by SAW flavors: **a** hot (CTmax > 0 °C), **b** top 10% hottest (CTmax > 7.8 °C), **c** cold (CTmax < 0 °C), and **d** top 10% coldest (CTmax < − 4.9 °C). Tmax anomaly, SLP and winds are shown in color shades, contours, and black arrows, respectively. The SoCal and the Great Basin domains are shown as in Fig. 2a

**Fig. 5:**
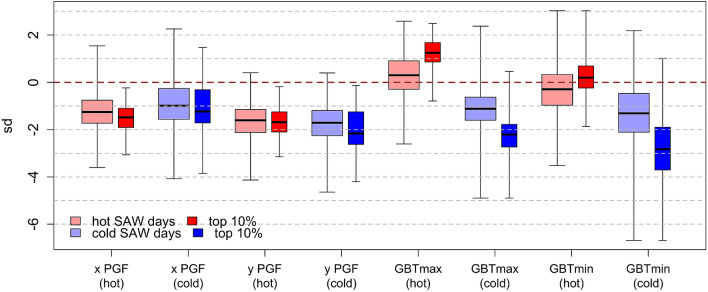
Box plots showing composites of standardized pressure gradient force (PGF); in zonal (x) direction and meridional (y) directions and Great Basin (GB) Tmax and Tmin anomalies conditional on hot and cold SAW days

Both hot and cold Santa Ana winds involve broad regions of high sea level pressure (SLP) over the interior Western U.S. (Figs. 2 and 4). This region of high SLP is centered approximately in northern Utah, on the northeastern edge of the Great Basin, for hot SAWs (Fig. 4a, b). The extreme hot SAW composite displays ~ 5 °C positive temperature anomalies in the northwestern GB and ~ 10 °C at the SoCal coast (Fig. 4b). During cold SAWs (Fig. 4c, d), the interior high pressure intensifies and expands, splitting into two centers: one in eastern Idaho/southwestern Montana and the other in the northwestern GB (northwestern Nevada/southeastern Oregon). Besides cold SoCal (negative anomalies of 5–10 °C), extreme cold SAWs start with cold Tmax anomalies down to nearly − 10 °C in the western GB (Fig. 4d). Tmin anomalies in the western GB show the greatest magnitudes, however (Fig. 5). Cold SAWs involve tighter SLP gradients extending into California and further north along the Sierra Nevada. In addition to temperature anomalies observed along the north and central California coast as well as the western slope of the Sierra Nevada (Figs. 2a, 4a, c), this provides evidence supporting coordination of SAWs—both hot and cold—with northern California’s Diablo winds (Smith et al. 2018a). In SoCal, the northeasterly SAWs and their associated PGF values turn slightly more northerly for cold compared to hot SAWs (Figs. 4, 5).

**Fig. 6.**
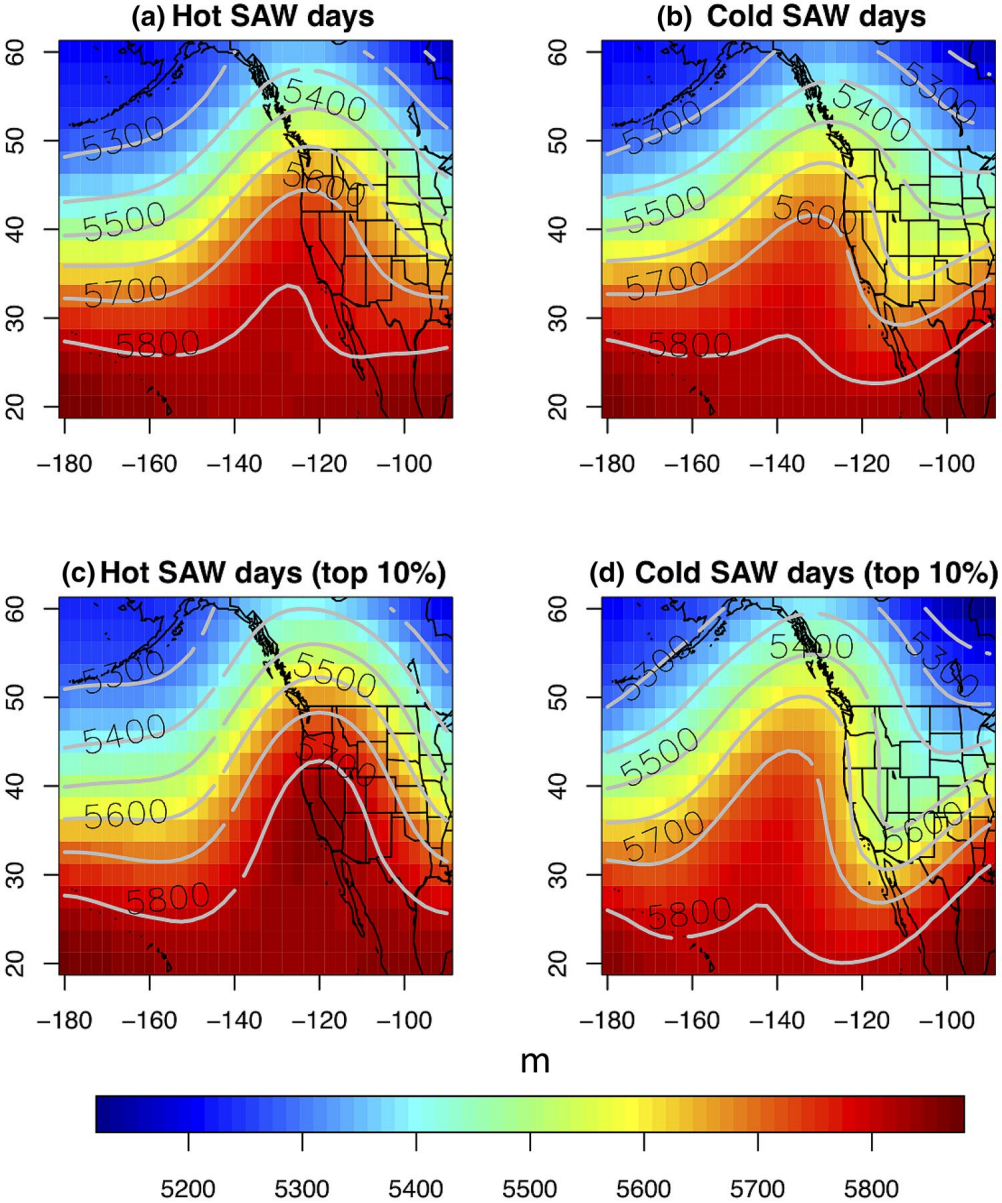
500 mb heights composited on hot and cold SAW days (**a**, **b**) and on top 10% hot and cold SAW days (**c**, **d**, respectively)

The synoptic scale upper-level circulation pattern conducive to cold SAWs displays a positively tilted ridge adjacent to the western coast of North America indicative of anticyclonic Rossby wave breaking (Ryoo et al. 2013) and strong baroclinicity (Fig. 6). Hot SAWs, however, appear to be associated with neutrally-tilted high amplitude flow around a blocking high centered at the California coast (Fig. 6). This behavior is stable from month-to-month across the SAW season (Figure S2) and suggests different synoptic causes for the two flavors of SAWs: cold SAWs associated with transient disturbances (short waves) and planetary wave breaking, whereas hot SAWs are linked to stationary planetary waves, specifically anticyclonic flow around a blocking high. The coldest SAWs tend to be driven by the strongest pressure gradients pushing northeasterly winds into SoCal (Figure S3), which tend to be somewhat stronger and more northerly than those associated with the weaker hot SAWs (Figs. 5 and S3).

### Southwestern precipitation and the Great Basin snow connection

Based on our dynamical interpretation, we expect widespread cold-frontal precipitation in the days preceding cold SAWs and dry conditions/limited precipitation preceding hot SAWs. This would be consistent with gradual Great Basin warming/abrupt cooling prior to hot/cold SAWs (Figures S4–S5). Nonetheless, negative Great Basin Tmin anomalies still occur during hot SAW days (Figure S5a). Figure S6 shows precipitation by month accumulated and composited over the five days preceding cold and hot SAWs. The entire Western US, including the GB and SoCal, accumulates precipitation on the days preceding cold SAWs. Hot SAWs are preceded by precipitation over the northwestern US, while the Southwest remains dry. This is the case throughout the SAW season, with the wet/dry contrast particularly pronounced during the most active SAW season (November–February).

**Fig. 7:**
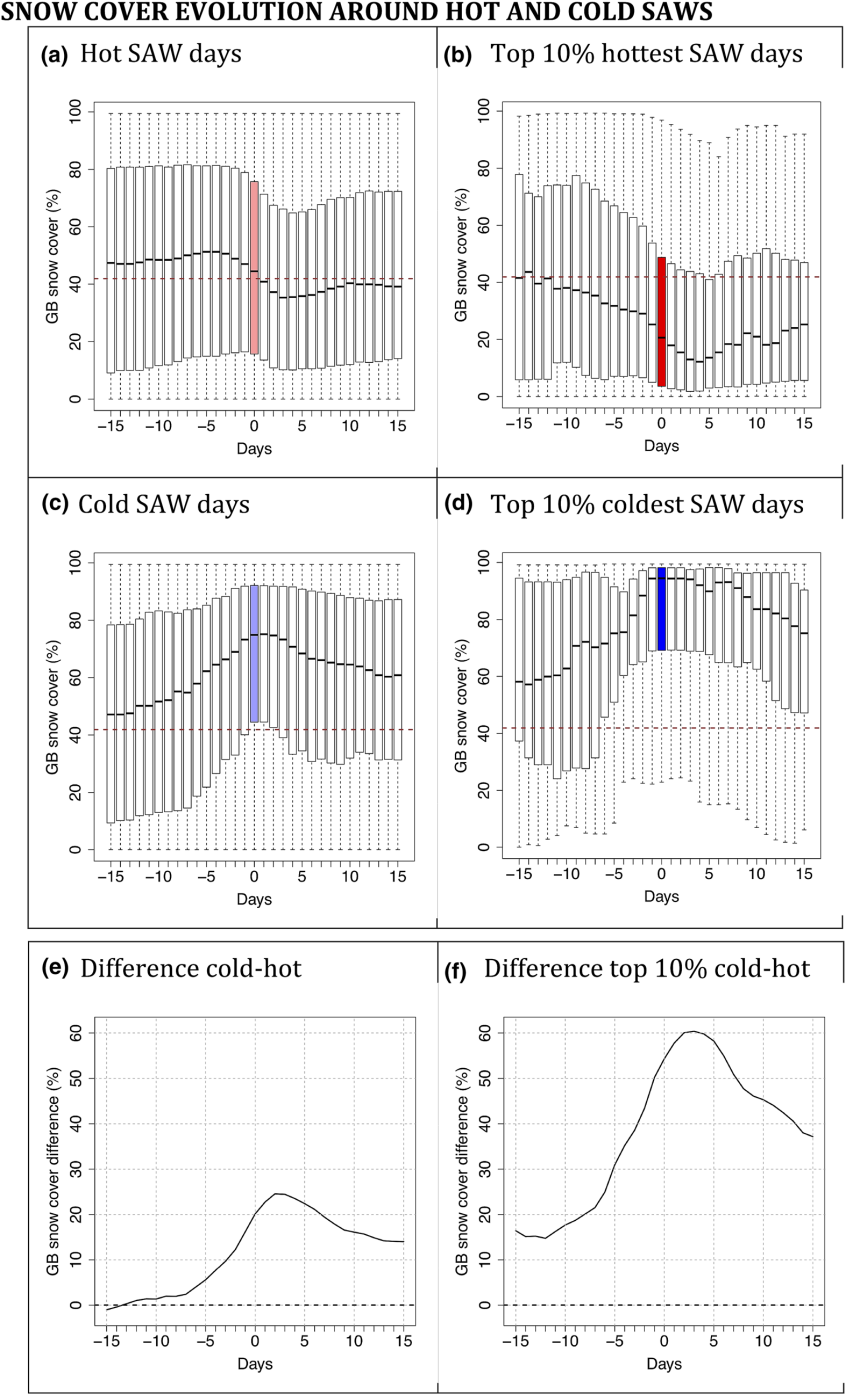
Great Basin snow coverage 15 days before and after SAW days. Thick black line marks the median, boxes lower and upper limit correspond to the 1st and 3rd quartiles, respectively, and whiskers extend to the farthest extreme values. Colored bars correspond to snow cover on SAW days (at x = 0, around which compositing was done) for each category, **a**–**d**. Red dashed line marks the percentage of snow coverage over the Great Basin domain (42%) averaged from November to January. **e**, **f** Show the differences of cold minus hot SAW days and top 10% cold minus hot SAW days, respectively

The majority of the Great Basin (i.e., areas aside from the highest mountains) is characterized by ephemeral snowpacks that accumulate during storms and later melt, a process that can happen multiple times during the cool season (Hatchett 2021). An element accentuating the difference between hot and cold SAWs is additional radiative cooling of airmasses during cold SAWs by the ephemerally snow-covered Great Basin. Indeed, composite evolutions of snow cover leading up to cold and hot SAWs indicates snow depletion/accumulation in advance of hot/cold SAWs (Fig. 7). The coldest SAWs tend to start with anomalous positive snow cover leading up to the event. However, the sample of extreme cold SAWs is severely reduced for this result as most cold SAW days occurred in the early half of the record, while the snow reanalysis data begins in October 1981.

Precipitation and snowpack data support the distinct synoptic setups leading to hot and cold SAWs. Great Basin ephemeral snow cover associated with these contrasting synoptic dynamics further explains part of the temperature differences between the two flavors of SAWs. These findings suggest the risk of wildfire should decline during cold SAWs, which tend to be preceded by wetting rains over SoCal (Figure S6) that increase fuel moisture. Moreover, the transient nature of synoptic disturbances associated with cold SAW dynamics should, in principle, make them shorter-lived than the hot SAWs associated with more stable and more persistent circulation regimes.

### Connection with wildfire

**Fig. 8:**
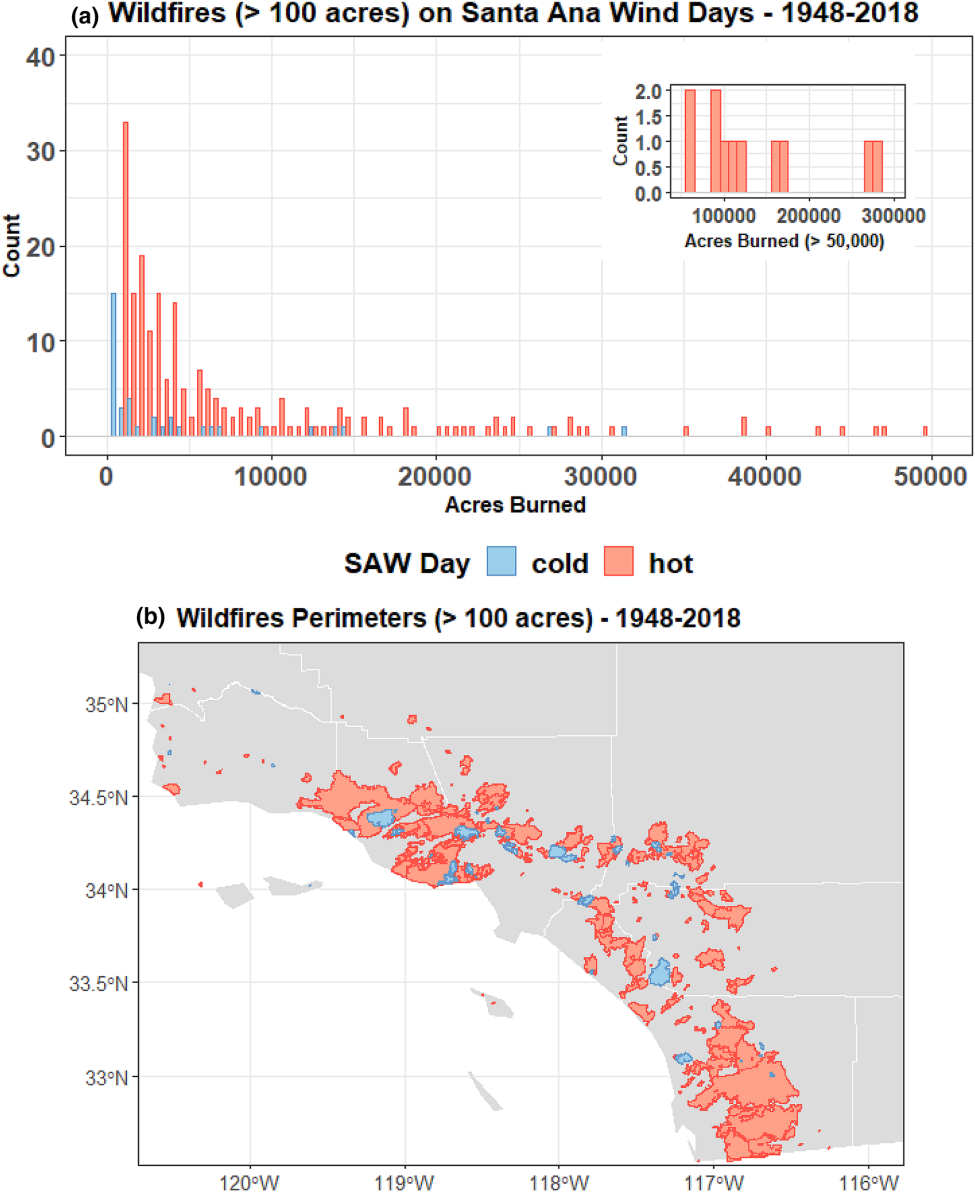
Histograms of acres burned (**a**) by wildfires in coastal SoCal that started during hot (red) and cold (blue) SAW episodes. Domain map (**b**) and fire perimeters associated with wildfires occurring during hot and cold SAW days

Although some of the strongest SAWs are of the cold variety (Fig. 3a), given the tendency of cold SAWs to be preceded by wetting rains and hot SAWs to be warmer, drier, and of longer duration (Figure S1), we expect larger wildfires in SoCal to be more frequently associated with hot SAWs. The data corroborate this expectation (Fig. 8). Acres burned by fires started during SAW conditions show that 90% of the large fires and 95% of the burned area occurred during hot SAWs. The maximum acres burned for a single cold SAW wildfire is 31,447; all wildfires exceeding this size ignited and grew during hot SAW episodes and commonly during extreme winds (SAWRI > 90th percentile). Because early-season cold SAWs are associated with little preceding precipitation, they can also fan already burning wildfires that have been ignited previously—this was the case in early September 2020, when an early-season cold SAW spread wildfires that started during extreme heat (not associated with SAWs) events of August.

### A note on long-term trends (1948–2018)

The interior Southwest is among the most rapidly warming regions of the contiguous US (USGCRP 2018). We therefore expect SAWs to reflect this inland warming, imprinting it episodically onto SoCal’s coastal region. CTmax displays seasonally (September–May) averaged warming trends amounting to 0.9 and 1.6** °C** over the entire seven-decade time period, respectively, for SAW and non-SAW-days (Fig. 2c). There are fewer SAW than non-SAW days resulting in smaller annual samples and greater variability of SAW compared to non-SAW CTmax. Moreover, the seasonal SAW CTmax index is biased towards December when SAW frequency is highest (GM’16).

**Fig. 9:**
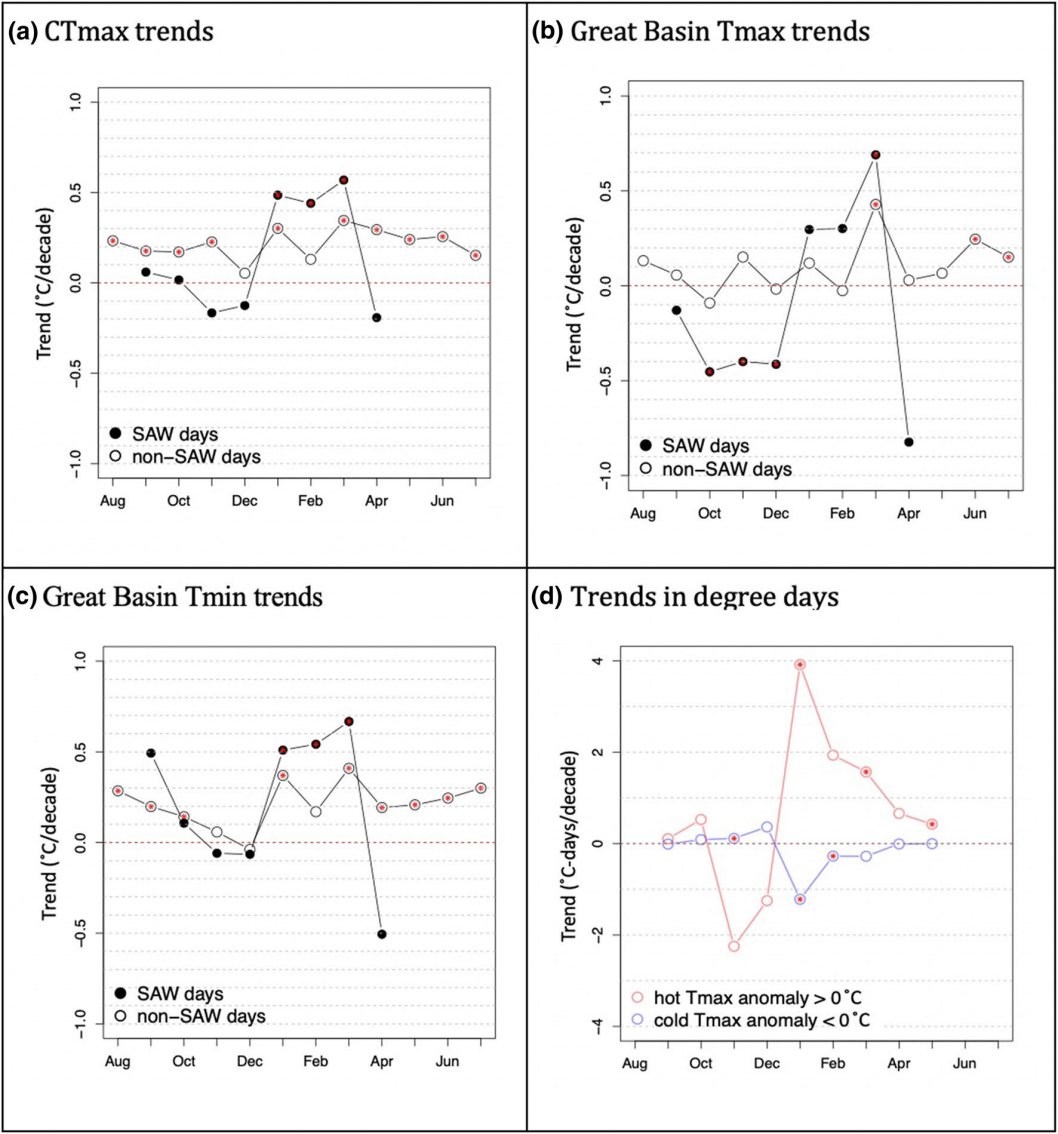
Tmax trends by month in °C per decade over the 71-year record averaged over SoCal (**a**) and the Great Basin Tmax (**b**) and Tmin (**c**). Monthly trends of cold and hot SAW “activity” measured in CTmax degree days (monthly sums of hot and cold CTmax excursions) that reflect both frequency and intensity of hot and cold SAWs (**d**). Red dots mark trends that are statistically significant with 95% confidence

Importantly, monthly trends in SAW versus non-SAW Tmax display very different seasonalities (Fig. 9). Over the 71-year record, coastal temperatures associated with SAWs have been significantly increasing in January, February and March (JFM; mean warming of ~ 3.5 °C). Weaker warming trends for non-SAW days were observed over SoCal with similar warming across months (Fig. 9a). Monthly temperature trends over the Great Basin (Fig. 9b, c) show the same seasonal pattern of warming, focused on JFM during SAW days and nights and were of comparable magnitude to the CTmax (SAW) trend. On the other hand, October-December (OND) trends are negative for SAW-associated Tmax over the Great Basin. Non-SAW Great Basin Tmax does not display significant trends except in March, June and July, while non-SAW Tmin trends are positive in all months except November, December and February. The strongest and most consistent warming is observed for SAW-associated Tmin over the Great Basin and CTmax (SAW) in JFM. Non-SAW Tmin warming is also strongest in January and March (Fig. 9c). March displays the most consistent warming trends observed over the SoCal coast and over the Great Basin (Tmax and Tmin) for both SAW and non-SAW days.

Exploring the seasonality of SAW-related CTmax trends, we have to consider what is causing the seasonality of warming in the Great Basin and the broader intermountain west. The timing and magnitude of SoCal and Great Basin trends is consistent with the strongest observed warming of inland temperatures, particularly in spring (Hoerling et al. 2013). This seasonality of western inland warming has been associated with declining snowpack and earlier snowmelt as part of spring’s progressively earlier start observed since the 1970’s (Cayan et al. 2001). With continued warming observed across all months over the Southwestern US, snow accumulation has been decreasing as more of the precipitation falls as rain (Knowles et al. 2006; Lynn et al. 2020). The warming, accentuated in spring, is also causing a tendency for snow to melt earlier (Mote et al. 2018). This likely leads to stronger regional warming through contemporaneous snow-albedo and delayed soil-moisture-related feedback mechanisms. It is noteworthy, however, that the Great Basin has not warmed as much as the surrounding interior Southwestern U.S., particularly in December, when it cooled, and in January (Figure S7). In February and March, GB warming was on par with the rest of the West. In March, Western U.S. warming has been substantial and widespread (Cayan et al. 2001). Figure S7 corroborates the robustness of this spring warming trend.

While the seasonal pattern of GB warming plays an important role, the breakdown of causes behind CTmax and GB Tmax/Tmin trends may differ by month. The relative activity (prevalence and intensity summarized in degree days—Fig. 9d) of warm vs cold SAWs appears to modulate the nature and seasonal structure of CTmax trends. During January and March we see significant positive and negative trends in both hot and cold SAW activity, respectively. February is the only other month that fits into this pattern, although not significantly so.

## Discussion and conclusions

Two distinct flavors of Santa Ana winds emerged from our analysis of SoCal coastal temperatures associated with SAWs: hot and cold. Extreme expressions of these SAW flavors have resulted in some of the hottest and coldest temperatures recorded in SoCal’s coastal zone in the past 71 years. These two flavors of SAWs result from very different synoptic setups and they impact wildfire hazard differently. Hot SAWs are associated with high-amplitude anticyclonic flow around blocking high-pressure systems centered over the California coast setting up a surface southwestward pressure gradient force directed from Nevada into California. The warming of the Great Basin starts a few days prior to and peaks a couple of days after SAW onset. In contrast, cold SAWs are set up by baroclinic anticyclonic Rossby wavebreaking associated with transient cold-frontal cyclones moving eastward across the West. Cold SAWs are preceded by widespread precipitation over the western US and accumulation of snow over the GB followed by a cold post-frontal airmass settling into the freshly snow-covered GB prior to the onset of cold SAWs. The surface high associated with this cold airmass is broader and extends further into the western GB, resulting in cold SAWs being somewhat more northerly then their warm northeasterly sisters. The strongest SAWs in terms of windspeed tend to be of the cold variety, while the driest and longest events tend to be of the hot variety.

The differing synoptic origins and land surface conditions favored by hot and cold flavors of SAW brings up important questions regarding the predictability and mesoscale dynamics involved in each SAW flavor. For instance, do the transient synoptic waves preceding cold SAW show less predictability compared to the quiescent anticyclonic conditions associated with hot SAW? How do observed mesoscale wind patterns vary between SAW types? Do hot SAW events tend to be gustier than cold SAWs by virtue of large-scale forcing favoring increased mesoscale gravity wave breaking? Are cold SAW events more likely to have flows channelized down canyons and through terrain gaps (Rolinski et al. 2019) due to their stronger katabatic (Hughes and Hall 2010) component? State-of-the-art reanalysis products such as ERA5 (Hersbach et al. 2020) are sufficient to broadly identify downslope winds (Abatzoglou et al. 2021), however their 25–50 km horizontal resolutions remain too coarse to capture the interactions of flow and terrain at the meso-γ scale (2–20 km; Thunis and Bornstein 1996). Answering these questions can be addressed by applying high-resolution numerical weather models to produce regionally downscaled climatologies (e.g., Hughes and Hall 2010; Rolinski et al. 2016; Smith et al. 2018a). Forecast skill metrics for both SAW flavors can be calculated and compared using a reforecast approach or by evaluating archived operational model output (e.g., High Resolution Rapid Refresh Model; Benjamin et al. 2016). Case studies following the approaches of Cao and Fovell (2016) and Fovell and Gallagher (2018) are also recommended to test the sensitivity of forecast skill and dynamical responses to varying land surface characteristics (e.g., snowpack conditions in the Great Basin).

Wildfires in SoCal are clearly partial to hot SAWs (Fig. 8). Although cold SAWs tend to be windier, they are usually preceded by precipitation over wind- and fire-prone coastal topography. Besides not being associated with precipitation over SoCal, hot SAWs tend to have lower relative humidity and are generally longer lasting. These differences result in 90% of the SAW-driven wildfires and 95% of the area burned being associated with hot SAWs. The impacts associated with hot SAWs extend beyond the immediate coastal heat, wildfire, and smoke. Many of SoCal’s mountains are highly susceptible to damaging and deadly post-fire debris flows (Oakley et al. 2017, 2018) and landslides (Rengers et al. 2020) for multiple years after wildfire has occurred. Changes in fire severity and intensity are also driving type conversions of native chaparral ecosystems towards grasslands, resulting in losses in biodiversity (Syphard et al. 2018).

In terms of downslope wind-driven anomalous heating patterns, both flavors of SAWs appear to be coordinated with other downslope wind regimes of California, including Sundowner winds of the northern Transverse Ranges (Smith et al. 2018b; Hatchett et al. 2018) and Diablo winds of Northern California (Smith et al. 2018a). This potential coordination needs to be studied in more detail to evaluate onset timing and seasonality differences and spatial extents and magnitudes of warming and wildfire occurrence. Besides identified regional-scale differences in hot and cold SAW direction, the interaction of circulations with local topography may amplify how these flavors of SAWs influence temperature and wind patterns differently. This can be studied with finer-resolved wind data and modeling experiments. However, the propensity of cold SAWs to be preceded by rain as well as their higher RH and shorter duration appear to notably diminish the fire hazard compared to the hot SAWs that are of longer duration, drier, and warmer.

The hottest ten SAW days have occurred throughout our 71-year record, while the coldest SAWs display a clear preference for the early decades. This is consistent with the strong seasonality of the observed trend in our coastal temperature index, the CTmax, which increased significantly in January, February and March, but not in other months. This JFM warming amounts to ~ 3.5 °C since mid twentieth century and almost twice the background (non-SAW) warming. It is produced by a commensurate seasonality of warming in the Great Basin during SAW events and a decrease/increase in cold/hot SAW activity comprising frequency and intensity. This change may be exacerbated by the gradual loss of snow cover in the Great Basin as part of a West-wide trend (Knowles et al. 2006; Mote et al. 2018). The JFM CTmax warming trend reflects the preference of the coldest SAWs for the 1940s–1960s. The hottest SAWs, occurring disproportionately in fall, when no significant long-term trends have been detected, are spread uniformly over the seven decades of record.

The new understanding of SAW flavors allows us to ask more informed questions about how SAWs will evolve amidst a warming climate to be addressed in future work. In view of these results, studies of future SAWs (Hughes et al. 2011; Miller and Schlegel 2006; Guzman Morales and Gershunov 2019) and consideration of future wildfire risk (Yue et al. 2014; Jin et al. 2015; Williams et al. 2019; Goss et al. 2020) focused on SoCal could be updated to resolve and incorporate trends in hot and cold flavors of SAWs, which are differentially related to wildfire. The diminishing SAW activity projected by GMG’19, specifically, will be revisited in future work to nuance those projections with respect to SAW flavors. The fuel-drying potential of warming SAWs should also be assessed. The winter/spring CTmax warming that has occurred already suggests an increasing potential of warmer and drier SAWs to dry out coastal vegetation and, particularly in anomalously dry winters, enhance the coastal wildfire season even into spring. Such spring wildfires have occurred in very hot SAWs in May 2014.^5^

This work was initially motivated by the public health impacts of SAW-driven coastal heat waves (Schwarz et al. 2020) and SAW-driven wildfire smoke (Leibel et al. 2019; Aguilera et al. 2020b, 2021a, b) in SoCal’s densely populated coastal zone. Impacts from heat waves and wildfires compounded in August and September 2020 to harm California during the preparation of this manuscript and prior to the traditional onset time of SoCal’s wind-driven autumn wildfire season. These events, further compounded and complicated by the SARS-CoV-2 pandemic, highlight the urgency of improving our understanding and prediction of the key weather ingredients that shape SoCal’s heat waves and wildfires. By documenting the underlying mechanisms that drive hot and cold SAWs and outlining the instrumental influence that SAWs exert on hazardous weather and fire extremes in SoCal, we hope that our results will inform the implementation of early warning systems to protect vulnerable coastal communities. As climate change bolsters both heat waves (Gershunov and Guirguis 2012) and wildfires (Williams et al. 2019; Goss et al. 2020) in California, evolving integrated early warning systems are urgently needed to mitigate risks to public health and to improve emergency preparedness to these increasingly prevalent risks. This work is a step towards that goal in California and other regions of the world with similar exposures and possibly even greater vulnerabilities to extreme weather events.

## Supplementary Information

Below is the link to the electronic supplementary material.

Supplementary file1 (DOCX 23966 kb)

## Data Availability

The Santa Ana wind regional index and coastal temperature indices used and produced in this article are available here: https://weclima.ucsd.edu/data-products/.

## Footnotes

1 https://www.climatespy.com/climate/summary/united-states/california/los-angeles-intl/september/1978.

2 https://www.latimes.com/california/story/2020-02-27/thursdays-heat-wave-might-break-some-records-in-southern-california. https://www.ocregister.com/2020/02/27/record-setting-pushes-into-southern-california-but-cooling-trend-on-its-way/.

3 https://abc7.com/southern-california-weather-cold-winds-freezing-temperatures/5902852/. https://www.latimes.com/california/story/2019-12-16/santa-ana-winds-and-cool-temperatures.

4 ERA5 data used in this work was downloaded from the ECMWF Copernicus Climate Data Store, https://doi.org/10.24381/cds.adbb2d47.

5 https://en.wikipedia.org/wiki/May_2014_San_Diego_County_wildfires

## Code availability

We will make any of our code used here available upon request.
