# Supplementary material for: Hot and cold flavors of southern California’s Santa Ana winds: their causes, trends, and links with wildfire
Source: Clim Dyn. 2021 May 31;57(7-8):2233–48. doi: 10.1007/s00382-021-05802-z (PMC8165508; doi:10.1007/s00382-021-05802-z)
Supplement: Supplementary file 1 — Supplementary file1 (DOCX 23966 kb) [file 382_2021_5802_MOESM1_ESM.docx]

**Hot and cold flavors of southern California’s Santa Ana winds:**

**Their causes, variability and trends**

**SUPPLEMENTARY FIGURES AND TABLES**

**ABSOLUTE HOTTEST AND COLDEST COASTAL TEMPERATURES ON RECORD**

| **a) Top 10 hot days** | | | | **b) Top 10 cold days** | | | |
| --- | --- | --- | --- | --- | --- | --- | --- |
| Date | Abs CTmax (˚C) | Abs CTmin (˚C) | R1D-SAWRI (m/s) | Date | Abs CTmax (˚C) | Abs CTmin (˚C) | R1D-SAWRI (m/s) |
| 09/27/10 | 39.48 | 17.56 | 0 | 01/11/49* | 6.23 | -2.51 | 0 |
| 07/06/18 | 38.10 | 17.01 | 0 | 01/10/49 | 6.65 | -3.63 | 0 |
| 09/02/55 | 38.04 | 18.99 | 0 | 01/28/57 | 7.18 | -2.24 | 0 |
| 09/01/55 | 38.03 | 18.87 | 0 | 01/29/57* | 8.03 | 1.20 | 0 |
| 09/26/63 | 38.02 | 18.24 | 0 | 01/27/57 | 8.15 | -0.28 | 0 |
| 09/01/17 | 38.00 | 19.69 | 0 | 12/14/67 | 8.15 | -1.85 | 5.10 |
| 09/24/78 | 37.98 | 16.05 | 3.23 | 12/10/72* | 8.17 | -0.65 | 0 |
| 07/07/18 | 37.73 | 21.02 | 0 | 12/22/90 | 8.18 | -4.27 | 6.36 |
| 09/27/63 | 37.53 | 17.72 | 0 | 01/22/62* | 8.24 | 0.87 | 0 |
| 06/16/81 | 37.44 | 14.26 | 2.29 | 01/04/49 | 8.51 | -3.88 | 6.41 |

**Table S1**. Top 10 coastal hottest and coldest days according to absolute coastal Tmax (CTmax) from 1948 to 2018. The corresponding absolute coastal Tmin (CTmin) values are also shown for each category. SAW conditions for those days are identified using SAWRI derived from the hybrid downscaling (R1D-SAWRI). In the top 10 cold days section, asterisk mark dates immediately preceding SAW events.

**HOTTEST AND COLDEST COASTAL TEMPERATURE ANOMALIES ON RECORD**

| **a) Top 10 hot days** | | | | **b) Top 10 cold days** | | | |
| --- | --- | --- | --- | --- | --- | --- | --- |
| Date | CTmax (˚C) | CTmin (˚C) | R1D-SAWRI (m/s) | Date | CTmax (˚C) | CTmin (˚C) | R1D-SAWRI (m/s) |
| 04/06/89 | 17.08 | 7.03 | 0 | 01/11/49 | -11.11 | -6.47 | 0 |
| 04/07/89 | 15.64 | 6.58 | 0 | 01/10/49 | -10.69 | -7.58 | 0 |
| 04/05/89 | 15.43 | 5.57 | 3.64 | 01/28/57 | -10.27 | -6.47 | 0 |
| 04/20/09 | 14.53 | 4.98 | 0 | 12/10/72 | -10.26 | -5.35 | 0 |
| 05/15/14 | 14.39 | 6.41 | 0 | 12/14/67 | -10.08 | -6.38 | 5.10 |
| 03/26/88 | 14.08 | 5.00 | 0 | 12/22/90 | -9.63 | -8.48 | 6.36 |
| 04/27/04 | 13.83 | 5.05 | 0 | 11/17/64 | -9.60 | -3.01 | 5.23 |
| 09/27/10 | 13.45 | 5.71 | 0 | 12/09/72 | -9.50 | -6.68 | 0 |
| 10/24/17 | 13.33 | 9.63 | 7.32 | 01/29/57 | -9.44 | -3.06 | 0 |
| 05/03/04 | 13.31 | 5.17 | 0 | 12/21/90 | -9.31 | -4.91 | 0 |

**Table S2**. Top 10 coastal hottest and coldest days according to de-seasonalized coastal Tmax (CTmax) from 1948 to 2018. The corresponding de-seasonalized coastal Tmin (CTmin) values are also shown for each category. SAW conditions for those days are identified using SAWRI derived from the hybrid downscaling (R1D-SAWRI). In the top 10 cold days section, asterisks mark dates immediately preceding SAW days.

| **(a)** SAW event duration   | **(b)** SAW event minimum RH   |
| --- | --- |

**Figure S1.**  Duration of SAW events (a), and minimum relative humidity (b) during SAW events. The extreme categories consider events when extreme SAW days have occurred.

**a) 500mb height composites on hot SAW days**

**
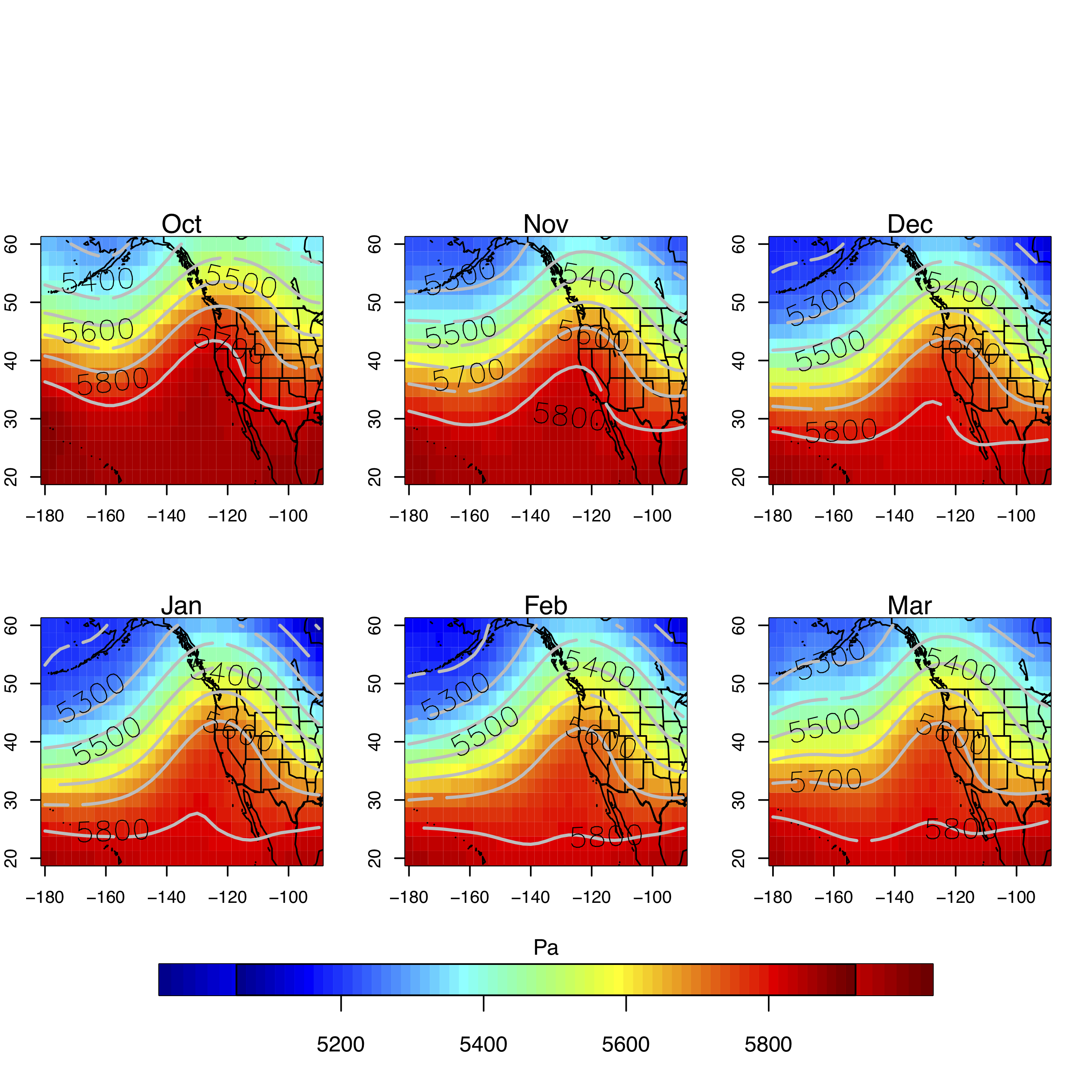
**

**b) 500mb height composites on cold SAW days**

**
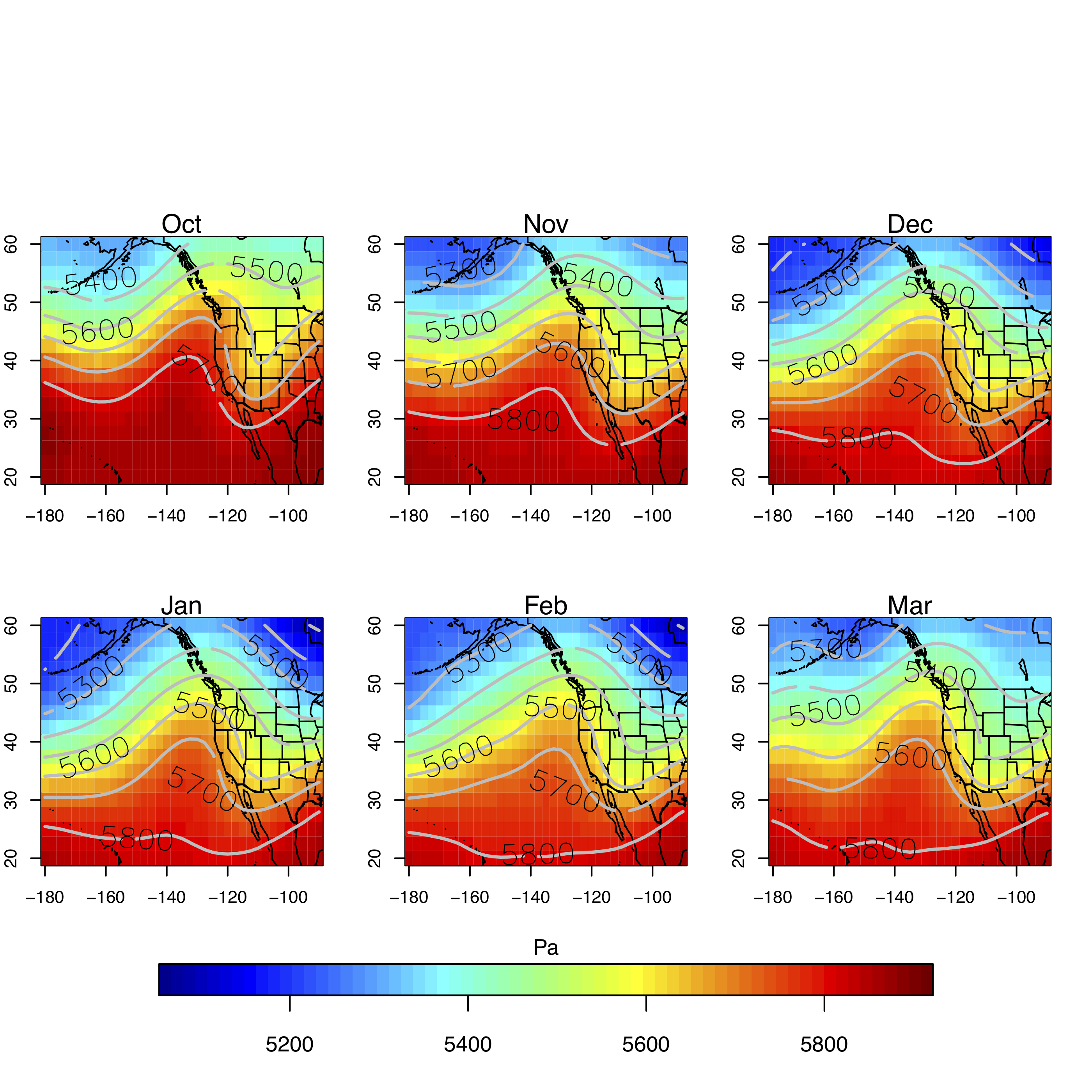
**

**c) Hot-cold SAW 500mb height composites differences**

**
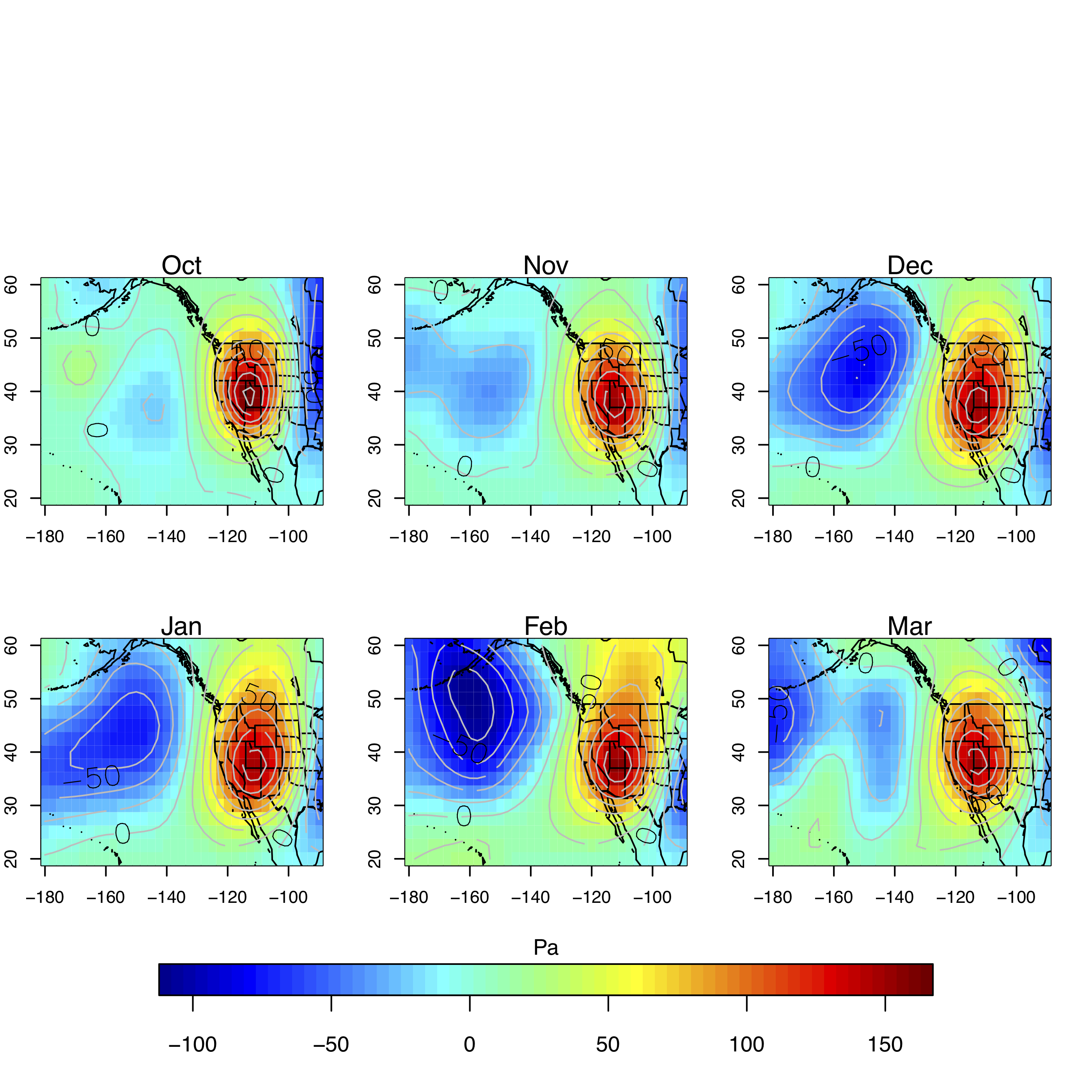
**

**Figure S2.** 500mb geopotential heights composited on hot and cold SAW days by months (panels a and b) hot-cold composites differences (panels c)

| **GB Tmax during SAW days**  **** | **GB Tmin during SAW days** |
| --- | --- |

**Figure S3.** Great Basin Tmax (a) and Tmin (b) as a function of the pressure gradient force (PGF) directed into SoCal (see Figure S2)

| **(a)** Hot SAW days   | **(b)** Top 10% hottest SAW days   |
| --- | --- |
| **(c)** Cold SAW days  **** | **(d)** Top 10% coldest SAW days  **** |
| **(e)** Difference cold-hot  **** | **(f)** Difference top 10% cold-hot  **** |

**Figure S4.** Great Basin Tmax 15 days before and after SAW days. Thick black line marks the median, boxes lower and upper limit correspond to the 1^st^ and 3^rd^ quartiles, respectively, and whiskers extend to the farthest extreme values. Colored bars correspond to snow cover on SAW days (at x=0, around which compositing was done) for each category, panels (a)–(d). Panels (e) and (d) show the differences of cold minus hot SAW days and top 10% cold minus hot SAW days, respectively.

| **(a)** Hot SAW days   | **(b)** Top 10% hottest SAW days   |
| --- | --- |
| **(c)** Cold SAW days  **** | **(d)** Top 10% coldest SAW days  **** |
| **(e)** Difference cold-hot  **** | **(f)** Difference top 10% cold-hot  **** |

**Figure S5.** Great Basin Tmin 15 days before and after SAW days. Thick black line marks the median, boxes lower and upper limit correspond to the 1^st^ and 3^rd^ quartiles, respectively, and whiskers extend to the farthest extreme values. Colored bars correspond to snow cover on SAW days (at x=0, around which compositing was done) for each category, panels (a)–(d). Panels (e) and (d) show the differences of cold minus hot SAW days and top 10% cold minus hot SAW days, respectively.

**a) Precipitation composites leading up to hot SAW days**

**
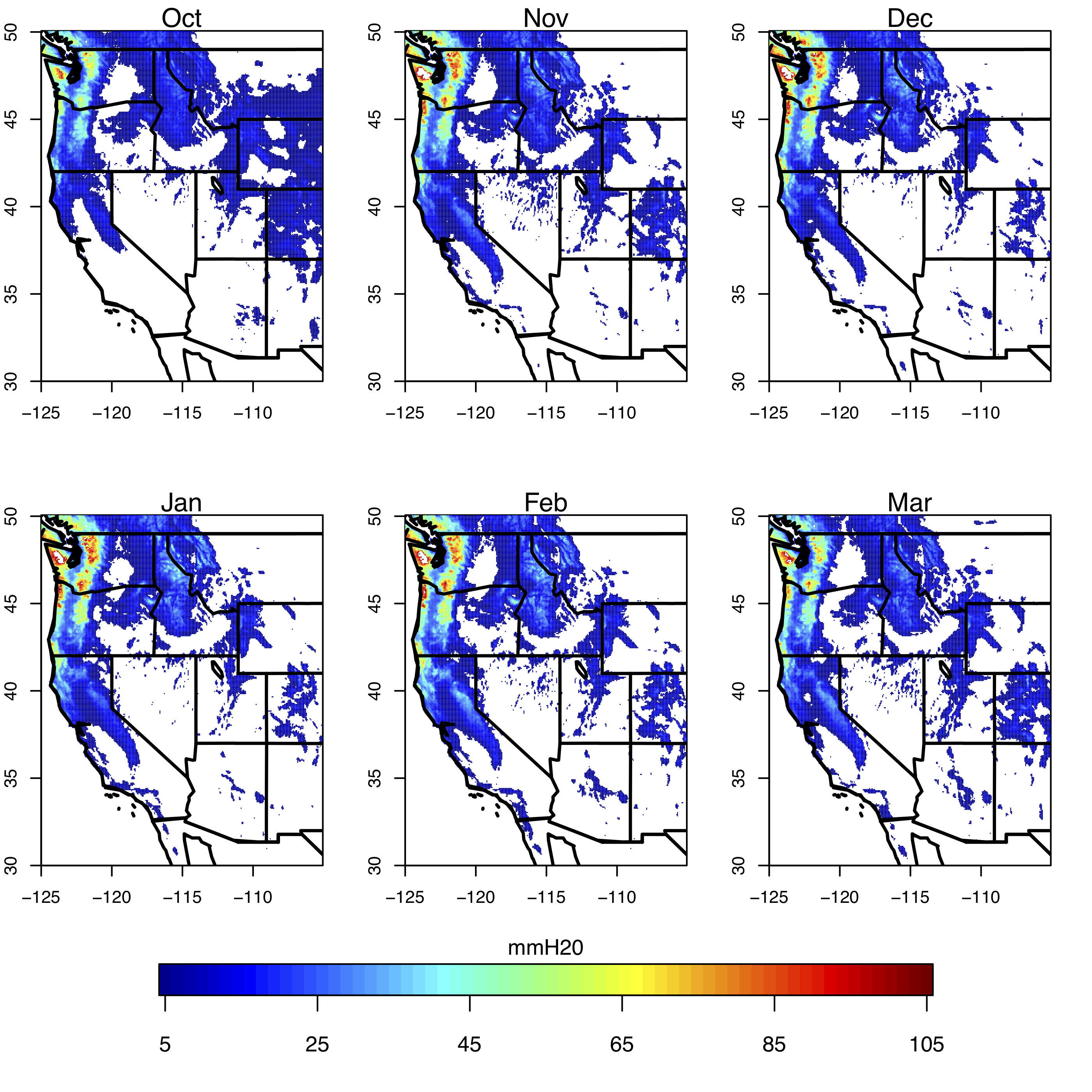
**

**b) Precipitation composites leading up to cold SAW days**

**
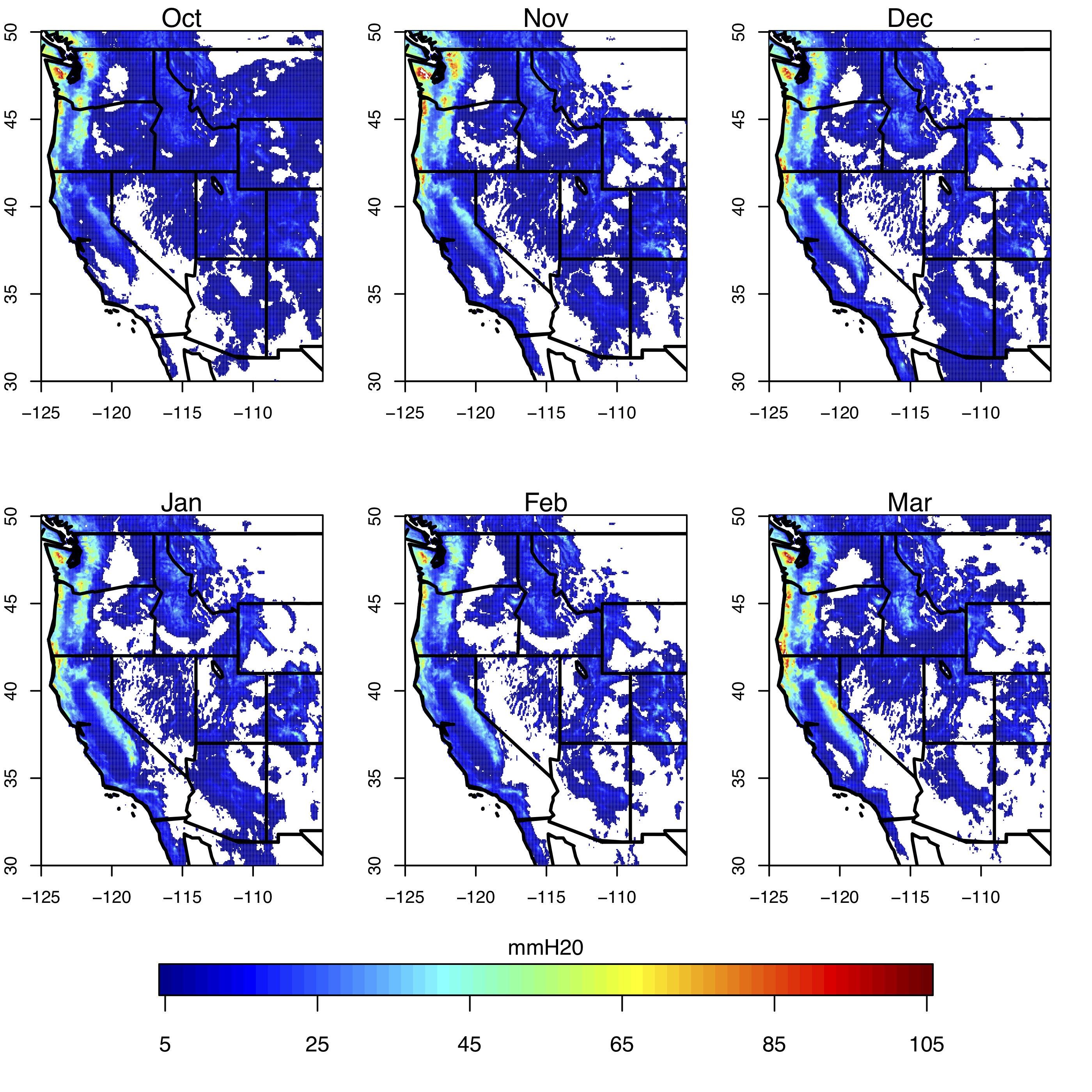
**

**Figure S6.** Precipitation composites by month accumulated over the five days leading up to hot (a) and cold (b) SAW days. Daily precipitation is from the Livneh et al. (2015) data set, which has been updated and spans 1950-2018.

| October Tmax  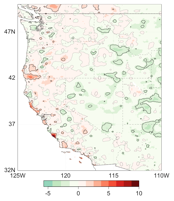 | November  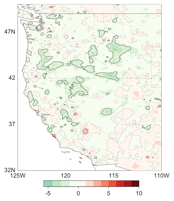 | December  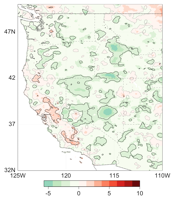 |
| --- | --- | --- |
| January  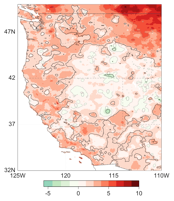 | February  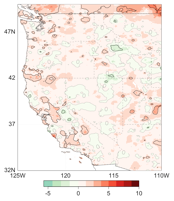 | March  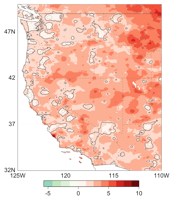 |

**Figure S7.** Linear trends in **°**C Tmax by month over the Western U.S. fitted locally over 1948-2018. Data are gridded daily Tmax observations from Livneh et al. (2015).
